# Sensitivity of Polyvoltine Thai Strains of *Bombyx mori* to a BmNPV Isolate From Mahasarakham

**DOI:** 10.1093/jisesa/ieaa023

**Published:** 2020-04-23

**Authors:** Siripuk Suraporn, Olle Terenius

**Affiliations:** 1 Department of Biology, Mahasarakham University, Thailand; 2 Department of Ecology, Swedish University of Agricultural Sciences (SLU), Sweden

**Keywords:** polyvoltine, *Bombyx mori*, nucleopolyhedrovirus, BmNPV-MSU, virulence

## Abstract

Virus infection by the *Bombyx mori* nucleopolyhedrovirus (BmNPV) is the most severe disease in Thai sericultural practice of polyvoltine silkworms. Here, we characterized a newly isolated BmNPV isolated from the Mahasarakham province in Thailand (BmNPV-MSU). The purity and morphology of BmNPV-MSU were examined using light microscopy and scanning electron microscopy. The polyhedral inclusion bodies (PIBs) of BmNPV-MSU appeared in tetragonal, hexagonal, octagonal, and globular forms. The virions were both single and multiple embedded as observed by transmission electron microscopy. We also determined the virulence of BmNPV-MSU for six different Thai polyvoltine strains by LC_50_ and time to death after infection. The LC_50_ values of Nang Lai, NK04, and Sam Rong strains were 5.05–1.52 × 10^7^ PIBs per ml and mortality peaked 7- to 8-d after inoculation. For Nang Noi, SP2, and RE05 strains the LC_50_ values were 7.91–1.82 × 10^6^ PIBs/ml and mortality peaked 4–5 d after inoculation, thus having lower chance of survival to infection by BmNPV-MSU.

Thailand has been known for its sericultural practice for centuries and Thai silk is an important export product. Thai silkworms (*Bombyx mori* L., Bombycidae, Lepidoptera) belong to the yellow race and are tiny but rich in sericin. Nowadays the silk is not only used for making silk cloth but it can also be used for other purposes. For instance, the fungus *Cordyceps*, which is important in traditional medicine, grows on silkworm pupae (Suraporn and Siriwattanametanon 2009).

One major problem of sericulture is diseases affecting *B. mori*. The most destructive disease in Thai sericulture is caused by the *B. mori* nucleopolyhedrovirus (BmNPV; Kumpratueang 1998). The infected silkworm expresses disease symptoms during the final stage of larval growth and dies without producing a cocoon resulting in the waste of time and labor for the farmer. It is believed that BmNPV lies dormant inside the silkworm, but can cause disease symptoms if activated by low temperature or certain chemicals such as hydrogen peroxide, potassium nitrate, or hydroxylamine (Himeno et al. 1973). The virus affects midgut epithelial cells, the tracheal system, hemolymph cells, the fat body, and the nuclei of middle and inner cells of the silk gland (Khurad et al. 2004).

BmNPV belongs to the genus *Alphabaculovirus* of the family Baculoviridae. The virus contains a circular double-stranded DNA with a molecular weight of 85,000 kDa. The baculovirus infection starts when a viral polyhedral inclusion body (PIB) is taken up perorally by the sensitive insect larva. Since the midgut of the lepidopteran larva is alkaline, it results in solubilization of the PIBs and release of virus particles into the alimentary system. The virus particles enter the midgut epithelial cells and are transported to the nuclei where they start the first cycle of viral production and replication. According to Smith-Johanssen et al. (1986), NPV infection is restricted to the nuclei of infected tissues. The evidence of NPV infection is hypertrophied nuclei, which almost fills the cells with numerous polyhedral bodies. Tracheal matrix and hypodermis tissue are heavily infected; moderate infection is found in the nuclei of silkglands and epithelial sheath of testes. There are no polyhedra in midgut cells, muscle cells, and malpighian tubules of silkworms (Smith-Johanssen et al. 1986). The infection causes many biochemical changes in the larva, which responds by changing the metabolism to defend itself against pathogen invasion.

Even though BmNPV is the key pathogen in sericultural practices in Thailand causing significant yield loss, there are no reports of Thai silkworm strains that are resistant to BmNPV. While a number of studies on virus resistance have been performed on bivoltine *B. mori* strains, very little work on NPV resistance and its virulence in polyvoltine silkworms has been performed. In this paper, we present data on a BmNPV collected in Mahasarakham (BmNPV-MSU) including morphology, virulence parameters, and effect on survival in different polyvoltine Thai *B. mori* strains.

## Materials and Methods

### Insects

We obtained silkworm eggs of six local Thai *B. mori* strains; Nang Lai, Nang Noi, NK04, RE05, Sam Rong, and SP2 from the Silk Innovation Center (SIC), Mahasarakham University (MSU), Thailand. The Thai silkworms Nang Lai and Nang Noi are native Thai silkworm strains while NK04, Sam Rong, SP2, and RE05 are hybrids (commercial strains). Newly hatched larvae were fed with mulberry leaves from trees cultivated at the Silk Innovation Center plantation. The silkworm larvae were reared under standard rearing conditions: 25–28°C, relative humidity 75–80%, and 12:12 h of light:darkness. Fresh mulberry leaves were offered to the silkworm larvae three times per day.

### Preparation of BmNPV

**Figure 1.**
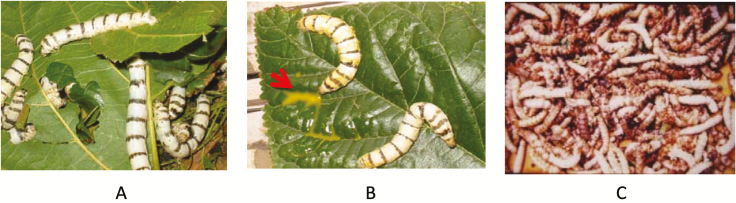
Thai native silkworm, *Bombyx mori*, Nang Lai. A) Healthy larvae, B) BmNPV-infected larvae exuding hemolymph (arrow), C) the cadavers of BmNPV-infected larvae in a severely diseased colony.

The BmNPV used in this study was originally isolated from infected silkworm larvae collected from a farmer’s rearing house in the Mahasarakham province, Northeastern Thailand. The virus was propagated in the Silkworm Diseases and Detection Laboratory, Silk Innovation Center, Mahasarakham University. Third-instar silkworm larvae of the native Thai silkworm Nang Lai were used as hosts for viral propagation. Silkworm larvae were infected by dipping mulberry leaves into BmNPV polyhedra suspended in distilled water. Three to five days after BmNPV inoculation, the larvae appeared yellowish and puffy and the hemolymph exuded from the wounds. To confirm the presence of polyhedra of BmNPV and their purity, the hemolymph from infected larvae was observed under a light microscope at 40×. The virus-infected larvae died within 4–6 d from when they started to show symptoms of disease. In contrast, the healthy silkworm larvae were strong and were eating normally, and had a clear hemolymph (Fig. 1a). Dead larvae were collected and homogenized in distilled water, and the homogenate was filtered through four layers of cheese cloth. The BmNPV polyhedra were pelleted by centrifugation at 1,400 × *g* for 10 min. The BmNPV polyhedra were observed under a scanning electron microscope. The concentration of PIBs was determined by hemocytometer count.

### Determination of BmNPV Virulence

The virulence of BmNPV was assayed by LC_50_ determination using six strains of third-instar *B. mori* larvae. The strains assayed were Nang Lai, Nang Noi, NK04, RE05, Sam Rong, and SP2. Before BmNPV oral inoculation, third-instar larvae were starved for 6 h. The larvae were fed mulberry leaves (2 × 2 cm) dipped in a BmNPV suspension at the concentration of 10^5^, 10^6^, 10^7^, 10^8^, 10^9^ PIBs/ml. For each concentration, three replicates of 35 larvae each were used (*n* = 105) and a control group (75 larvae) was fed mulberry leaves dipped in distilled water. After BmNPV treatment, the larvae were allowed to feed fresh mulberry leaves three times a day (morning, afternoon, and evening). Every day, the mortality was counted and dead larvae were removed. To determine the median lethal concentration (LC_50_) of BmNPV, we used the ToxRat Standard Version 3.2.1 (ToxRat Solutions GmbH, 2005). The time to death after infection was also recorded as an estimation of BmNPV virulence.

### Sequence Analysis

To determine the relationship of BmNPV-MSU to the previously isolated BmNPV-Thailand (Zhou et al. 2012), the *polh* and *bro* genes were amplified and sequenced. PCR was run on DNA samples acquired from infected tissue following the protocols of Tang et al. (2017) using the primers found in Table 1. The PCR conditions were as follows: 94°C for 3 min, followed by 30 cycles of [94°C for 30 s, 58 to 48°C for 30 s (the temperature was decreased by 1°C in every cycle for 10 cycles and then held at 48°C for 20 cycles), and 72°C for 1 min], and followed by a final extension step at 72°C for 20 min. PCR products were sequenced at Macrogen (the Netherlands) and deposited in GenBank MN653955–MN653958.

**Table 1.** PCR primer pairs (from Tang et al. 2017)

Target gene	Primer sequence
polh	F: ATGCCGAATTATTCATACACCC R: TTAATACGCCGGACCAGTG
bro-a	F: ATGGCTCAAGTTAAAATTGG R: TTACAAGTTAAAATTGTTATTC
bro-b	F: ATGGCTCAAGTTAAAATCGGGC R: TTAGTTTTGCGAGCAGTGGGGC
bro-c	F: ATGGCTCAAGTTAAAATTGG R: TGCTTAAACGCTTGACGACATA
bro-d	F: TATTGCGCCGCAGGAAGCCAT R: CGAATCGTCACGCGTCGTTGTA
bro-e	F: ATTAGTTTTGCGAGCAGTG R: ATGGCTCAAGTTAAAATTGG

## Results and Discussion

BmNPV infections cause great damage to the silk production (Attathom and Sinchaisri 1987, Edneia et al. 2006). It is the most severe disease in the Thai silkworm industry and BmNPV outbreaks may occur throughout the year, but particularly during the summer season and the rainy season (Kumpratueang 1998, Dandin et al. 2000).

The isolated BmNPV-MSU was purified from silkworm larvae collected from silkworm rearing in the Mahasarakham Province, Thailand. In our study, no symptom was observed during the first 2- to 3-d after infection. With the progress of the disease, infected larvae lost appetite and were characterized by swollen abdominal segments. At this stage, the various tissues like epidermis, fat bodies, and hemocytes were damaged. The larvae appeared creamy white to yellowish in color and shrunken, the integument became fragile, ruptured upon touch, releasing a viscous liquid containing viral polyhedra (Fig. 1b). Three to five days after virus feeding, the larvae showed typical symptoms of BmNPV infection. The nucleopolyhedrovirus MSU isolate was highly pathogenic to silkworm larvae and exhibited pronounced characteristic symptoms in the fifth-instar larvae just before or at the time of cocoon formation (Fig. 1c) and consequently the crop loss was 100%. The larvae died within 4–6 d since the time point they started to show symptoms of disease.

**Figure 2.**
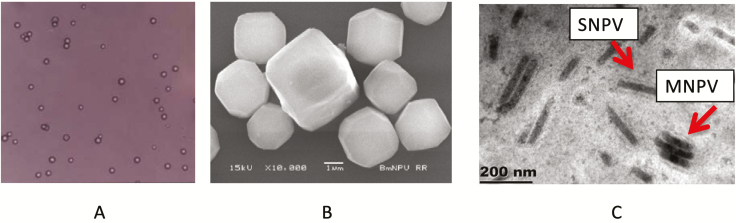
The BmNPV–MSU forms polyhedral inclusion bodies. A) Observed under light microscope (40×), B) tetragonal, hexagonal, octagonal, and globular polyhedral inclusion bodies observed under a scanning electron microscope, C) most virus particles are single-embedded BmSNPV while some are multiple embedded BmMNPV (arrows).

The BmNPV polyhedra were collected from the hemolymph and the purity was determined by light microscopy. At 40× magnification, the PIBs appeared as small spots with circles (Fig. 2a). The presence of BmNPV polyhedra was confirmed by scanning electron microscopy. The morphological forms of BmNPV-MSU polyhedra were tetragonal, hexagonal, octagonal, and globular (Fig. 2b). The nucleocapsids (virions) were packed in PIBs and had two morphological features: either one nucleocapsid located in an envelope, so-called single embedded (BmSNPV) or more than two nucleocapsids multiply embedded (BmMNPV; Fig. 2c). In comparison, Attathom and Sinchaisri (1987) found that the morphology of PIBs from BmNPV, also collected in Northeastern Thailand, only had a tetragonal shape and virions of the BmSNPV type. The BmNPV-MSU was isolated more recently than the published BmNPV-Thailand (Zhou et al., 2012). To make a comparison with the BmNPV-Thailand, we amplified the *polh*- and *bro* genes. The *polh* gene of BmNPV-MSU was identical with that of BmNPV-Thailand; however, the *bro* genes were slightly different. For *bro-a*, the nucleotide sequence identity was 99% between BmNPV-MSU and BmNPV-Thailand, for *bro-c* the identity was 90% and for *bro-d* it was 98%. In contrast to BmNPV-Thailand, we found a *bro-b* gene in BmNPV-MSU (GenBank MN653955).

**Figure 3.**
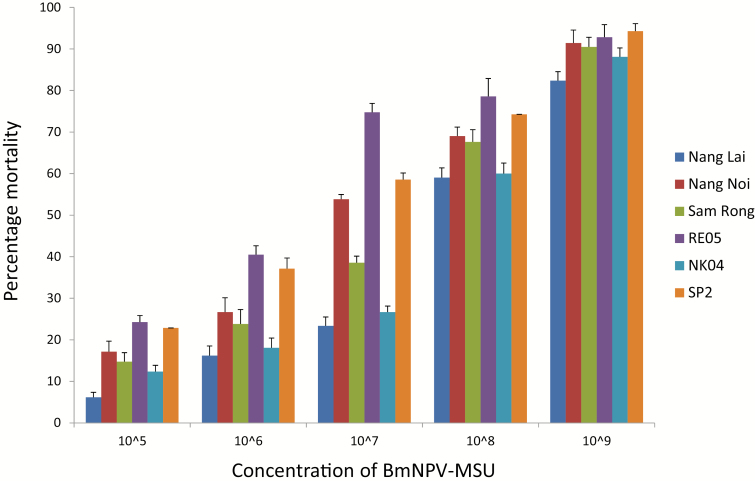
Percentage of dead larvae after feeding different concentrations of BmNPV. Error bars indicate standard deviation of three replicates.

The virulence of BmNPV was quantified in six strains of Thai silkworm measuring survival ratio at different concentrations, and time to death after a single dose. The strains Nang Lai, Nang Noi, NK04, RE05, Sam Rong, and SP2 were inoculated per os in various concentrations: 10^5^_,_ 10^6^_,_ 10^7^_,_ 10^8^, and 10^9^ PIBs/ml. The mortality ratio of BmNPV at 10^7^ PIBs/ml was between 23% and 75% with the survival ratio in the order Nang Lai > NK04 > Sam Rong > Nang Noi > SP2 > RE05 (Fig. 3). In comparison to previous data on susceptibility to BmNPV-infection in bivoltine strains, the polyvoltine *B. mori* strains show a similar level of susceptibility. For example, Cheng et al. (2014) determined the level of susceptibility to BmNPV in four Chinese bivoltine strains: A35, A40, A53, and P50. They found that the LC_50_ for the most resistant strain A35 was 5.90 × 10^7^ PIB/ml and for the most susceptible strain P50 1.03 × 10^5^ PIB/ml. For the Thai strains used in our study, the susceptibility ranged from an LC_50_ for the most resistant strain Nang Lai of 5.05 × 10^7^ PIB/ml to an LC_50_ for the most susceptible strain RE05 of 1.82 × 10^6^ PIB/ml (Table 2). The high-producing strains NK04 and Sam Rong were introduced to the farmers as they were more resistant to BmNPV; with LC_50_ values of 1.52 × 10^7^ and 3.13 × 10^7^, respectively, they are among the most resistant strains of the six strains tested.

**Table 2.** LC_50_ value of BmNPV for Thai *Bombyx mori* strains in order of resistance

LC_50_ (PIBs/ml)	5.05 × 10^7^	3.13 × 10^7^	1.52 × 10^7^	7.91 × 10^6^	3.54 × 10^6^	1.82 × 10^6^
Insect strains^*a*^	Nang Lai	NK04	Sam Rong	Nang Noi	SP2	RE05

^*a*^The experiments were performed in triplicates with 35 larvae per experiment.

**Figure 4.**
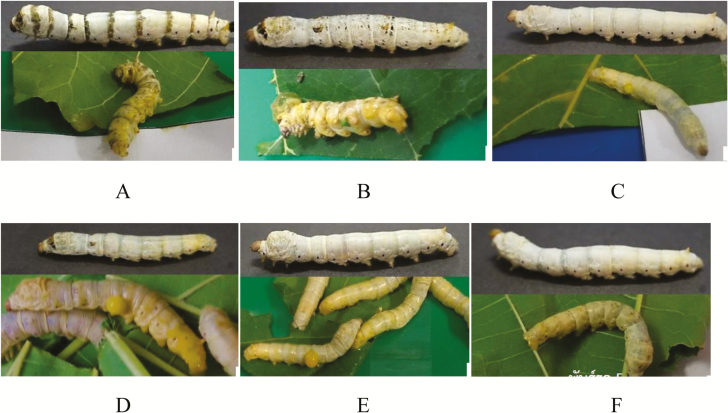
The appearance of diseased Thai silkworm fifth-instar larva after 7 d of BmNPV-MSU inoculation. Severely infected larvae appear yellowish in body color and subsequently diseased larvae show turbid hemolymph containing BmNPV polyhedra exuded from the wounds. Control larvae upper part, infected larvae lower part; A) Nang Lai, B) Sam Rong, C) NK04, D) SP2, E) Nang Noi, F) RE05.

We also analyzed the survival after feeding a single-dose BmNPV-MSU. Twenty silkworm larvae of each strain were fed 10^7^ PIBs/ml BmNPV-MSU and death was recorded daily (Table 3). The results showed that the strains can be divided into two groups where Nang Noi, SP2, and RE05 had a higher loss of larvae as compared to the strains Nang Lai, NK04, and Sam Rong. The group with higher death rate also started to die earlier (Table 3). The higher sensitivity to BmNPV-MSU infection was manifested in more severe disease symptoms for Nang Noi, SP2, and RE05 (Fig. 4).

**Table 3.** Time to death for *Bombyx mori* larvae infected by BmNPV- MSU

Silkworm strain (*n* = 20)	Days after BmNPV-MSU inoculation (10^7^ PIBs/ml)									Number of dead larvae
	1	2	3	4	5	6	7	8	9	
Nang Lai				1		2	3	2		8
NK04						1	1	5	1	8
Sam Rong						1	4	2		7
Nang Noi			2	5	3	1				11
SP2			1	3	6	3	1			14
RE05			1	6	2	1	2			12

In this paper, we have presented how the baculovirus BmNPV-MSU differentially affects polyvoltine Thai *B. mori* strains. Our further investigation will focus on understanding the mechanism behind the observed differences in survival.

## References

[CIT0001] Attathom T., and Sinchaisri N.. 1987 Nuclear polyhedrosis virus from *Bombyx mori* in Thailand. Sericologia. 27: 287–295.

[CIT0002] Cheng Y., Wang X.Y., Chang D., Gao J. and Xu J.P.. 2014 Expression of several antiviral related genes to BmNPV in different resistant strains of silkworm, *Bombyx mori*. J. Insect Sci. 14: 1–9. 10.1093/jis/14.1.76 PMC4212868 25373223

[CIT0003] Dandin S.B., Jayaswal J., and Giridhar K.. 2000 Handbook of sericulture technologies. Central Silk Board, Bangalore, India.

[CIT0004] Edneia F.B., Torquato H.M., Miranda N. D., Rose M.C., Valdeni B., and Franco S.. 2006 Systematics, morphology and physiology of nucleopolyhedrosis virus: scanning electron microscopy technique. Neotrop. Entomol. 35: 787–789. 17273710 10.1590/s1519-566x2006000600011

[CIT0005] Himeno M., Matsubara F., and Hayashiya K.. 1973 The occult virus of nuclear polyhedrosis virus of silkworm larvae, *Bombyx mori*. J. Invert Entomol. 22: 436–445.

[CIT0007] Khurad A. M., Mahulikar A., Rathod M. K., Rai M. M., Kanginakudru S., and Nagaraju J.. 2004 Vertical transmission of nucleopolyhedrovirus in the silkworm, *Bombyx mori* L. J. Invertebr. Pathol. 87: 8–15. 15491594 10.1016/j.jip.2004.05.008

[CIT0008] Kumpratueang S. 1998 Development of a DNA probe for early detection of grassery disease of silkworm, *Bombyx mori*. M.Sc. thesis. Kasetsart University, Bangkok,Thailand, 129 p.

[CIT0009] Smith-Johannsen H., Witkiewicz H., and Iatrou K.. 1986 Infection of silkworm follicular cells with *Bombyx mori* nuclear polyhedrosis virus. J. Invertebr. Pathol. 48: 74–78.

[CIT0010] Suraporn S., and Siriwattanametanon W.. 2009 Growth of *Cordyceps* spp. on the pupae of Thai silkworm, *Bombyx mori*, Nanglai X 108. J. Kamphaengsean Academic, Kasetsart University, Thailand. 7: 1–9 [in Thai].

[CIT0011] Tang F., Zhang Y., Shaoa Y., Zhua F., Huanga P., and Baia X.. 2017 Isolation and identification of a new *Bombyx mor*i nucleopolyhedrovirus strain isolated from Yunnan, China. ScienceAsia 43: 26–32.

[CIT0012] Zhou J. B., Li X. Q., De-Eknamkul W., Suraporn S., and Xu J. P.. 2012 Identification of a new *Bombyx mori* nucleopolyhedrovirus and analysis of its *bro* gene family. Virus Genes. 44: 539–547. 22311430 10.1007/s11262-012-0721-1

